# Socioeconomic status and educational inequality: digital competency pathways to creative problem-solving and academic performance

**DOI:** 10.3389/fpsyg.2025.1690989

**Published:** 2026-01-15

**Authors:** Jinglu Liu, Yuwei Chen

**Affiliations:** 1 Faculty of Education, Open University of China, Beijing, China; 2 Lifelong Education Research Institute, Open University of China, Beijing, China

**Keywords:** creative problem-solving, digital competency, digital divide, digital technology, educational inequity, socioeconomic status

## Abstract

**Introduction:**

The digital divide extends beyond technology access to create educational inequalities through differential competency development, where socioeconomic advantages enable sustained digital engagement that enhances creative problem-solving. Grounded in social cognitive theory and digital divide frameworks, this study examines how socioeconomic status (SES) influences student outcomes through competency-mediated pathways that enhance creative problem-solving and academic performance.

**Methods:**

Two studies in China examined competency pathways using structural equation modeling. Study 1 analyzed PISA data (*N* = 16,148) measuring SES, digital use, digital competency, and creative problem-solving. Study 2 (*N* = 558) additionally assessed academic ranking.

**Results:**

Higher SES influenced digital use, which further led to digital competency, ultimately enhancing creative problem-solving. Additionally, digital use affected both academic ranking and creative problem-solving, while digital competency selectively enhanced creative problem-solving only, suggesting traditional academic assessments may not capture the strategic skills that constitute digital competency.

**Discussion:**

Findings demonstrate how SES-based resource disparities create differential competency acquisition in digital learning environments, providing cumulative advantages in creative problem-solving. This dual-pathway model supports social cognitive theory by showing how SES shapes competency development through digital use behaviors, while advancing digital divide theory through sequential mechanisms across multiple levels. These findings provide practical guidance for educational institutions to collaborate with parents in designing programs that foster digital competency development through sustained technology engagement for higher-order cognitive outcomes.

## 1 Introduction

Competency development represents one of the fundamental processes underlying educational success, as students who acquire stronger competencies consistently demonstrate superior academic outcomes and improved life prospects (Gosselin et al., 2013). Recent educational psychology research emphasizes that competency acquisition occurs through complex interactions between environmental resources, behavioral engagement, and personal cognitive factors, making it crucial to understand how different learning contexts facilitate or constrain this developmental process (Abedini et al., 2024; Li and Xue, 2023).

Digital learning environments have emerged as particularly important contexts for competency development, as technological proficiency increasingly plays a central role in students’ ability to access learning opportunities and demonstrate their skills. From a psychological perspective, digital competency encompasses both technical skills and the confidence to apply these skills strategically in learning situations (Getenet et al., 2024; Wang et al., 2021). This competency development process operates through social cognitive mechanisms, where environmental advantages facilitate observational learning and self-efficacy development, ultimately enhancing students’ academic performance (Bandura, 1986; Bandura, 1997).

Social cognitive theory provides a robust framework for understanding how socioeconomic resources translate into competency advantages through psychological pathways. Higher-SES Students benefit from increased access to digital learning environments, providing enhanced opportunities for observational learning, guided practice, and competency development (Rosales-Márquez et al., 2025). Digital divide theory offers a complementary structural framework, describing how inequalities progress sequentially from basic access (first-level) to sophisticated usage patterns (second-level) and meaningful educational outcomes (third-level) (Scheerder et al., 2017; van Deursen and van Dijk, 2019).

While research has documented relationships between SES and digital competencies (Kuo-Hsun, 2021; Tondeur et al., 2010), and between digital skills and academic performance [Organisation for Economic Co-operation Development [OECD], 2020], a critical gap exists in understanding the sequential psychological mechanisms linking all three levels of the digital divide. Previous studies have largely examined direct associations rather than testing the sequential progression from first-level access through second-level competency development to third-level educational outcomes. Moreover, existing research has not sufficiently distinguished between qualitatively different types of digital engagement. Recent longitudinal evidence reveals that general informal exposure to digital media (such as videos, print materials, songs, and everyday oral communication) predicts learning gains, whereas structured academic activities like tutorial classes and homework completion show weak or even negative associations (Tsang and Lo, 2025). This suggests that the type of digital engagement may matter as much as the amount, with different activity types potentially operating through distinct psychological mechanisms. Additionally, contrary to traditional assumptions, associations between socioeconomic status and informal digital exposure appear weaker than expected, likely because low-cost platforms have democratized access to certain forms of digital content (Tsang and Lo, 2025). These findings motivate a more nuanced examination of how forms of digital use translate into competency development and educational outcomes.

Our study addresses these significant theoretical gaps by providing an empirical investigation of dual-pathway mechanisms operating across all three levels of the digital divide. First, we test sequential competency development mechanisms, examining how socioeconomic resources relate to digital use (first-level), which subsequently leads to digital competency (second-level), ultimately corresponding to educational outcomes (third-level). Second, we provide a granular examination of dual pathways, distinguishing between procedural skills developed through digital use versus strategic metacognitive skills acquired through digital competency. Third, we test whether these distinct pathways lead to different student outcomes through different psychological mechanisms.

The Chinese educational context provides an ideal natural laboratory for examining these mechanisms, as Chinese educational systems are undergoing rapid technological transformation through massive government investments in digital infrastructure nationwide (Hashim et al., 2022). Moreover, the highly competitive, examination-oriented system with its emphasis on standardized testing creates distinct pressures and incentives for technology use that differ substantially from Western educational models, offering unique insights into digital competency development processes.

To ensure methodological rigor of our theoretical model in this distinctive context, we employ a two-study design using structural equation modeling with independent Chinese samples. Study 1 utilizes large-scale PISA data (*N* = 16,148) to establish foundational sequential competency mechanisms and dual-pathway associations within this high-stakes academic environment, while Study 2 employs primary survey data (*N* = 558) to cross-validate these findings and examine the additional objective outcome, namely student academic ranking. This methodological approach provides both replication strength and complementary evidence, enhancing confidence in the psychological mechanisms underlying digital competency development while addressing a fundamental question with profound implications for educational institutions: How do socioeconomic advantages translate into educational outcomes through sequential digital competency development, and what can educational institutions do to optimize these mechanisms for their students?

## 2 Literature review

### 2.1 Theoretical framework

Our study draws on two complementary theoretical perspectives, namely social cognitive theory and digital divide theory, to explain how SES shapes educational outcomes through sequential competency development pathways. Social cognitive theory (Bandura, 1986) provides a comprehensive psychological framework for understanding how competencies develop through interactions between environmental factors, behavioral engagement, and personal cognitive processes. Within this framework, competency acquisition occurs through observational learning, where students learn by watching others use digital technologies effectively, and through self-efficacy development, where confidence in one’s digital skills influences subsequent learning behaviors (Bandura, 1997; Schunk and Usher, 2019). Digital competency can be conceptualized as domain-specific self-efficacy, that is students’ confidence in their ability to organize and execute digital actions required to achieve educational goals (Ulfert-Blank and Schmidt, 2022).

The process of competency development follows social cognitive mechanisms through environmental-behavioral-personal interactions. Higher-SES students benefit from enhanced environmental resources that provide increased opportunities for observational learning and guided practice with digital technologies (Njeri and Taym, 2024; van Deursen and Helsper, 2015). This increased exposure facilitates behavioral engagement through more frequent and sophisticated digital use, which subsequently develops personal cognitive factors including digital self-efficacy and strategic competencies (Lopez-Garrido, 2023). Importantly, these competencies become self-reinforcing through reciprocal determinism, where enhanced digital confidence leads to increased engagement, further developing skills in an upward spiral of competency growth (Bandura, 1986).

Complementing social cognitive theory, digital divide theory has evolved beyond simple access issues to recognize multiple interconnected levels of digital inequality that align with competency development stages (Scheerder et al., 2017; van Deursen and van Dijk, 2019). This multi-level framework distinguishes between first-level access to digital technologies, second-level development of digital skills and usage patterns, and third-level differential educational outcomes derived from technology use. From a psychological perspective, these levels represent sequential stages of competency acquisition: initial exposure and familiarization (first-level), skill development and confidence building (second-level), and strategic application for educational advantage (third-level) (van Deursen and van Dijk, 2019). The integration of these frameworks reveals how socioeconomic differences translate into digital competency advantages through psychological mechanisms. Environmental resources associated with higher SES facilitate both greater frequency of digital use (behavioral factor) and exposure to educationally-relevant digital practices through observational learning opportunities. This increased and more sophisticated digital engagement develops digital self-efficacy and strategic competencies (personal factors), which function as cognitive resources that enhance educational outcomes. Crucially, this process creates dual pathways: procedural skills developed through frequent use versus strategic competencies acquired through sophisticated application, each operating through distinct psychological mechanisms to influence different types of educational outcomes.

### 2.2 SES and students’ creative problem-solving

Creative problem-solving represents a complex higher-order cognitive ability that requires students to identify novel problems, generate innovative solutions, and implement strategic approaches when confronting unfamiliar challenges (Adeoye and Jimoh, 2023). This cognitive skills have become increasingly important in educational contexts, as students must navigate complex academic tasks that demand flexible thinking and adaptive problem-solving strategies. Research consistently demonstrates that higher-SES students exhibit higher creative problem-solving compared to their peers from lower socioeconomic contexts, with these differences emerging across diverse assessment contexts and age groups (Duncan et al., 2017). This relationship exists because higher-SES families provide fundamentally different learning environments that foster creative thinking development. Specifically, these environments offer enhanced cognitive stimulation through diverse educational experiences, access to enrichment activities that challenge conventional thinking, and regular exposure to sophisticated problem-solving models (Fraillon et al., 2020; Chmielewski, 2019). From a social cognitive perspective (Bandura, 1997), students in these environments develop stronger self-efficacy beliefs regarding their skills to tackle complex cognitive challenges, which subsequently encourages more persistent and strategic approaches to creative problem-solving tasks.

Digital learning environments present a more complex dynamic that potentially amplifies existing socioeconomic advantages in creative problem-solving development. Higher-SES students typically possess stronger foundational digital literacy skills from early home exposure, demonstrate greater confidence in exploring unfamiliar technological tools, and have developed more sophisticated strategies for information evaluation and synthesis through prior experiences (Afzal et al., 2023). Indeed, the quality and sophistication of digital resources available to students varies dramatically by socioeconomic background. Research demonstrates that low-income families lack access to the technology required for quality online education, while higher-SES families typically engage with premium educational platforms that offer superior learning experiences (Afzal et al., 2023). These premium resources provide interactive simulations, adaptive learning algorithms, and sophisticated problem-solving demonstrations that model creative thinking strategies more effectively than basic educational websites (Haelermans et al., 2020). Furthermore, higher-quality digital platforms offer real-time adaptive feedback systems and personalized learning pathways that can scaffold students’ development in complex problem-solving tasks. However, this depends also critically on students’ existing skills to leverage these tools effectively, a capacity that itself reflects prior socioeconomic advantages in educational preparation, digital literacy, and access to technical support systems (Haelermans et al., 2020).

### 2.3 SES, digital use, and digital competency

From a social cognitive theory perspective, digital use and digital competency development are shaped by environmental resources, behavioral engagement, and personal cognitive factors through reciprocal determinism (Bandura, 1997). Specifically, digital use represents students’ frequency of exposure to digital technology for educational purposes, while digital competency reflects their psychological self-efficacy in using digital devices. While digital use is influenced by environmental resource availability and access opportunities, digital competency develops through observational learning opportunities and self-efficacy building experiences that vary systematically across socioeconomic contexts, creating differential pathways for both technological exposure and competency acquisition (Schunk and Usher, 2019).

Specifically, SES shapes students’ digital use through differential access to technological resources and opportunities for educational engagement. Research consistently demonstrates that higher-SES students have greater access to digital devices, high-speed internet connectivity, and diverse technological resources, leading to more frequent and varied digital engagement for educational purposes (Rideout and Katz, 2016). These students typically engage with digital technologies more regularly both at home and in educational settings, benefiting from unlimited or less restricted access that allows for sustained engagement with digital tools and platforms. In contrast, lower-SES students often experience limited access characterized by shared devices, inconsistent internet connectivity, and time-restricted usage, resulting in significantly lower frequency of digital use for educational activities (Gonzales et al., 2020). This disparity in environmental resources translates directly into differences in usage patterns, with higher-SES students demonstrating more consistent and extensive digital technology engagement across educational domains.

SES also influences digital competency development through the quality of learning environments and observational learning opportunities available to students. Higher-SES Students typically demonstrate more advanced digital skills, because their environments provide superior modeling and scaffolding for complex digital practices (Livingstone et al., 2017). Higher-SES families are likely to provide guidance, instruction, and modeling of effective digital practices, creating environments that facilitate the development of digital self-efficacy and strategic competencies through social cognitive mechanisms (Hammer et al., 2021; van Deursen and Helsper, 2015). These students are more likely to receive formal and informal digital literacy instruction, observe sophisticated technology use by family members and peers, and have access to diverse software applications that promote advanced skill development. Conversely, lower-SES students often face multiple barriers to developing sophisticated digital competencies, including not only limited access to technology but also fewer opportunities for observational learning, less exposure to effective digital practices, and limited access to mentorship or instruction in advanced digital skills (Raihan et al., 2025). These environmental differences create notable disparities in the development of digital competencies through differential exposure to modeling, practice opportunities, and confidence-building experiences. Based on the above theoretical framework and empirical evidence, we formulated the following hypotheses:

*H1a*: SES is positively associated with students’ digital use.

*H1b*: SES is positively associated with students’ digital competency.

### 2.4 Digital use and digital competency

Digital competency development occurs through systematic engagement with digital technologies, where repeated exposure and practice facilitate the acquisition of both technical skills and psychological confidence in digital environments (Tzafilkou et al., 2022). Specifically, frequent digital use provides increased opportunities for observational learning as students encounter diverse technological practices, while sustained engagement builds self-efficacy through mastery experiences that enhance confidence in digital skills (Schunk and Usher, 2019). The iterative nature of digital interaction enables students to develop both procedural knowledge about technological operations and strategic competencies for complex problem-solving within digital contexts (Blanc et al., 2025; Organisation for Economic Co-operation Development [OECD], 2025). Ma and Ismail (2025) found that sustained digital engagement in educational settings significantly predicted students’ advanced digital competencies, particularly in areas requiring strategic thinking and information evaluation. Similarly, recent large-scale studies indicate that students who engage more frequently with educational technologies demonstrate superior digital self-efficacy and more sophisticated approaches to technology-mediated problem-solving tasks (Cha et al., 2025; Getenet et al., 2024). This relationship operates through cumulative learning processes where each digital interaction provides opportunities to refine technical skills, develop strategic approaches, and build confidence through successful task completion (Fraillon et al., 2020). Furthermore, frequent engagement allows students to observe and internalize effective digital practices through both direct experience and vicarious learning from digital interfaces and peer interactions, creating a self-reinforcing cycle of competency development (Godsk and Møller, 2025). This process transforms initial behavioral engagement with technology into sophisticated psychological competencies that transfer across diverse digital learning contexts. Therefore, we formulate the following hypothesis:

*H2*: Digital use is positively associated with students’ digital competency.

### 2.5 Digital use and students’ creative problem-solving

In digital contexts, technology serves as both a cognitive tool and medium for enhanced thinking processes (Kong and Wang, 2023, 2024). Digital use enhances creative problem-solving by expanding students’ exposure to diverse ideas and approaches beyond traditional learning environments (Kong and Wang, 2024). The key mechanism lies in how frequent digital engagement broadens intellectual horizons through continuous exposure to varied perspectives, multimedia content, and interactive experiences for innovative solutions (Kong and Wang, 2023). This develops ideational fluency, which is posited as a broader repertoire of concepts and strategies students can draw upon when confronting novel problems (Runco and Acar, 2012). Researchers suggest that digital environments create learning opportunities that traditional settings cannot easily replicate. For instance, Beghetto and Kaufman (2014) demonstrate that digital platforms facilitate mini-c creativity, that is personally meaningful creative insights emerging through interaction with diverse digital content. These environments increase opportunities for unexpected discovery of new tools, resources, and collaborative connections that spark creative thinking (Merchant, 2013). Moreover, the cognitive benefits actually extend beyond content exposure. For instances, repeated interaction with digital interfaces reduces mental effort required for technology navigation, freeing cognitive resources for creative rather than technical problem-solving (Sweller et al., 2019). This automation allows students to allocate more mental capacity to higher-order thinking tasks (Sweller et al., 2019; van Merriënboer and Sweller, 2010). Additionally, digital environments can usually provide immediate feedback and iterative opportunities supporting flexible thinking patterns essential for creative problem-solving (Barana et al., 2021; Karrer et al., 2023). Based on the above literature review, we therefore formulate this hypothesis:

*H3*: Digital use is positively associated with students’ creative problem-solving.

### 2.6 Digital competency and students’ problem-solving

Digital competency facilitates creative problem-solving by enabling students to synthesize information from multiple digital sources, developing broader knowledge bases that support creative thinking (Fraillon et al., 2020). Competent users can effectively employ simulation and visualization tools to test hypothetical solutions and explore alternative approaches that would be impossible in traditional learning environments (Mishra and Koehler, 2006). Furthermore, their ability to navigate collaborative digital platforms allows them to engage in collective problem-solving processes, exposing them to diverse perspectives and solution strategies that enhance creative thinking capacity (Conneely et al., 2020; Pifarré and Vendrell, 2022). The International Computer and Information Literacy Study has documented positive associations between students’ digital competency levels and their performance on creative problem-solving assessments, while PISA results indicate that higher digital competency correlates with stronger collaborative problem-solving performance, even after controlling for socioeconomic background (Organisation for Economic Co-operation Development [OECD], 2020). Past studies further reveal that students with advanced digital competencies maintain these advantages over time, with effect sizes ranging from moderate to large across different problem-solving domains (Li and Oon, 2024; Li et al., 2025).

Digital competency also enhances metacognitive awareness in problem-solving contexts. Students with higher digital competencies demonstrate better ability to monitor their problem-solving progress, evaluate solution effectiveness, and adjust strategies when using digital tools (Kong et al., 2019). Research by Goldhammer et al. (2016) shows that digitally competent students employ more sophisticated self-regulation strategies when navigating complex digital problem-solving environments. Their enhanced ability to engage with digital learning resources increases both motivation and self-directed learning skills, which in turn contribute to academic success (Song and Bonk, 2016). Past meta-analyses confirm these relationships, showing consistent positive effects of digital competency on both creative problem-solving and academic achievement across diverse educational contexts (Cheung and Slavin, 2013). Therefore, the following hypotheses are formulated:

*H4*: Digital competency is positively associated with students’ creative problem-solving.

In summary, the above literature review establishes three interconnected pathways through which SES influences students’ creative problem-solving. The evidence demonstrates that SES shapes both digital use (H1a) and digital competency (H1b), while digital use facilitates competency development through experiential learning (H2). Both digital use (H3) and digital competency (H4) independently enhance creative problem-solving through distinct mechanisms. Based on this literature and building on all these previous hypotheses, we therefore propose the following mediation hypotheses in our study:

*H5a*: Digital use mediates the relationship between SES and students’ creative problem-solving.

*H5b*: Digital competency mediates the relationship between SES and students’ creative problem-solving.

*H5c*: Digital use and digital competency sequentially mediate the relationship between SES and students’ creative problem-solving.

## 3 PISA study

### 3.1 Sample participants

Study 1 utilized data from the Programme for International Student Assessment (PISA) 2022. PISA data was selected due to its established reliability and validity in educational research, with extensive scholarly applications across diverse research questions and methodological approaches (Lewis, 2014).

The PISA 2022 dataset is particularly valuable for investigating digital competency among secondary school students as it comprehensively captures students’ digital access, in-school and out-of-school digital experiences, cognitive aspects of technology use, and family backgrounds. The dataset includes extensive information on school environments and technological contexts, making it well-suited for examining relationships between SES, digital skills, and academic outcomes within the Chinese educational context.

We extracted the Chinese sample from the PISA dataset, consisting of 16,148 students. The gender distribution was approximately balanced: 7,824 female students (48.5%) and 8,324 male students (51.5%). Participants spanned grades 7–12, with the majority in grade 9 (30%) and grade 10 (66.2%), reflecting typical grade placement of 15-year-old students.

### 3.2 Measures

#### 3.2.1 Socioeconomic status

SES was assessed using a composite index constructed from four individual items: family financial evaluation, annual family income, father’s education level, and mother’s education level. These items were standardized and weighted to derive a comprehensive SES score. Example items include: “How would you describe your family’s financial situation?” and “What is your mother’s highest level of education?” We specified a single-factor model in Mplus Version 10, with our four SES indicators loading onto one latent SES factor. Model parameters were estimated via robust maximum likelihood, and fit was evaluated against conventional thresholds (CFI and TLI ≥ 0.90, RMSEA ≤ 0.06, SRMR ≤ 0.08) to ensure an acceptable measurement model (Hu and Bentler, 1999). Upon confirming good fit, we extracted individual factor scores, yielding a continuous SES composite that accounts for each indicator’s unique contribution while modeling measurement errors (DiStefano et al., 2009). The SES measurement model demonstrated acceptable fit in both Study 1 [χ^2^(2) = 180.038, CFI = 0.957, TLI = 0.871, RMSEA = 0.075] and Study 2 [χ^2^(2) = 102.739, CFI = 0.969, TLI = 0.908, RMSEA = 0.067]. Standardized factor loadings ranged from 0.368 to 0.767 in Study 1 and from 0.402 to 0.859 in Study 2, with all loadings significant at *p* < 0.001. Reliability was 0.693 for Study 1 and 0.727 for Study 2.

#### 3.2.2 Digital use

Digital use was measured using a 7-item self-report scale assessing the frequency of students’ engagement with various digital devices and platforms for learning in school settings. Students rated how often they used different technologies using a 4-point Likert scale (1 = Never, 2 = Rarely, 3 = Sometimes, 4 = Often). Sample items included: “How often do you use at school: Educational software, games or apps, other learning tools,” and “How often do you use at school: A learning management system or school learning platform (e.g., Blackboard^®^, Edmodo^®^, Moodle^®^, Google^®^ Classroom™).”

#### 3.2.3 Digital competency

Digital competency was measured using a 14-item self-report scale. Students rated their proficiency in performing various digital tasks using a Likert scale (1 = I cannot do this, 4 = I can easily do this). Sample items included: “Search for and find relevant information online,” “Create a multi-media presentation (with sound, pictures, or video),” and “Identify the source of an error in software after considering a list of potential causes.”

#### 3.2.4 Creative problem-solving

Creative problem-solving was measured through a 10-item scale assessing students’ self-reported their creative thinking and problem-solving. Items were rated on a Likert scale (1 = Not at all confident, 4 = Very confident). Example items included: “Coming up with creative ideas for school projects,” “Inventing new things,” and “Addressing social problems like pollution.”

#### 3.2.5 Control variables

Several demographic and school-level variables were included as controls in our analyses to account for their potential influence on the key dependent variables. Student gender was coded as a binary variable (0 = male, 1 = female). Student grade was included to control for developmental differences across different academic years. We also included the education level of both students’ parents. These control variables were included in our model analyses to isolate the unique contributions of predictor variables on student outcomes while accounting for potentially confounding factors.

### 3.3 Results

#### 3.3.1 Preliminary analysis

Examination of missing data patterns and distributional characteristics informed our analytical approach. While most variables had low to moderate missing rates (SES: 2.4%; digital use: 4.12%; digital competency: 5.8%; creative problem-solving: 3.7%). Additionally, our assessment of normality revealed that most study variables demonstrated acceptable levels of skewness and kurtosis. To address the missing data, we employed Full Information Maximum Likelihood (FIML) in our analyses, which provides robust parameter estimates and standard errors while making use of all available data points (Enders, 2001; Muthén and Muthén, 2017).

To examine relationships among the key study variables, we conducted correlation analyses, summarized in Table 1. Digital use was positively correlated with digital competency (*r* = 0.219, *p* < 0.01) and creative problem-solving (*r* = 0.140, *p* < 0.01). In addition, digital competency demonstrated a strong positive correlation with creative problem-solving (*r* = 0.303, *p* < 0.01). For control variables, SES was significantly and positively correlated with both mother’s (*r* = 0.844, *p* < 0.01) and father’s education levels (*r* = 0.803, *p* < 0.01). Cronbach’s alpha values indicated strong internal consistency for digital use (0.87), digital competency (0.94), and creative problem-solving (0.93), supporting the reliability of these measures.

**TABLE 1:** Study 1 means, standard deviations, scale reliabilities, correlations, Cronbach’s reliability in parentheses.

Variables	Mean	SD	1	2	3	4	5	6	7	8
1. Grade	9.629	0.573	–							
2. Gender	1.515	0.500	-0.046**	–						
3. Mother education	4.233	1.105	0.086**	-0.005	–					
4. Father education	4.271	1.088	0.086**	-0.013	0.432**	–				
5. SES	3.078	0.447	0.003	-0.027**	0.844**	0.803**	***(0.69)***			
6. Digital technology use	2.903	0.883	0.053**	0.064**	0.026**	0.031**	0.132**	***(0.87)***		
7. Digital competency	3.171	0.666	0.009	-0.070**	0.049**	0.072**	0.099**	0.219**	***(0.94)***	
8. Creative problem-solving	2.523	0.673	-0.026**	0.066**	0.050**	0.063**	0.088**	0.140**	0.303**	***(0.93)***

*N* = 16,148 was used for correlation analysis. *M* = Mean, SD = Standard Deviation. SES is the composite score of the father’s education. The mother’s education, financial evaluation, and family annual income, calculated by saving the CFA factor scores of the four measuring items.

**p < 0.01; *p < 0.05.

#### 3.3.2 Construct validity

To establish the construct validity of our study variables, we conducted a comprehensive series of confirmatory factor analyses (CFA). Given the clustered nature of the PISA data, with students nested within schools, and schools within cities, we utilized TYPE = COMPLEX in Mplus for CFAs. This approach employs a sandwich estimator to adjust standard errors and test statistics for the non-independence of observations, preventing the artificial deflation of standard errors that would otherwise occur when ignoring the nested structure (Muthén and Muthén, 2017).

To assess convergent validity, we evaluated the measurement model for digital use, digital competency, and creative problem-solving. Given the extensive number of indicators (31 items), we employed item parceling in the overall CFA to maintain model parsimony and ensure estimation stability (Little et al., 2002). Using the item-to-construct balance approach (Little et al., 2013), we created balanced parcels by combining high and low loading items from individual CFAs: 4 parcels for digital competency, and 3 parcels for digital use and creative problem-solving. This more parsimonious measurement model demonstrated good fit [χ^2^ (32) = 3108.584, CFI = 0.951, TLI = 0.931, RMSEA = 0.078]. All parcel loadings were substantial and significant (ranging from 0.636 to 0.955, all *p* < 0.001), and reliability coefficients exceeded 0.85 for all constructs (digital use α = 0.87, digital competency α = 0.94, creative problem-solving α = 0.93), supporting the convergent validity of our constructs.

The three latent factors showed moderate positive intercorrelations: digital use with digital competency (*r* = 0.198, *p* < 0.001), creative problem-solving with digital competency (*r* = 0.323, *p* < 0.001), and creative problem-solving with digital use (*r* = 0.163, *p* < 0.001), offering the preliminary empirical distinctiveness of the constructs. To further establish discriminant validity, we conducted competing model analyses comparing our hypothesized three-factor model (M1) against increasingly parsimonious alternatives. We systematically tested a series of nested models: two-factor models (M2-M4) and single-factor models (M5; Table 2). Fit indices consistently favored the three-factor model, with substantial deterioration in model fit observed as factors were combined. These results provide evidence for the discriminant validity of our key study variables. Additionally, we also conducted the full measurement model for robustness check (see Supplementary Appendix B).

**TABLE 2:** Study 1 Discriminant validity for the three study variables.

Models	χ 2	df	CFI	TLI	RMSEA
M1: default 3-factor (DU, DC, CPS)	3108.584	32	0.951	0.931	0.078
M2: 2-factor (DU+DC, CPS)	19670.365	34	0.687	0.586	0.192
M3: 2-factor (DU, DC+CPS)	16847.571	34	0.732	0.645	0.177
M4: 2-factor (DU+CPS, DC)	20212.331	34	0.678	0.574	0.194
M5: 1-factor (DU+DC+CPS)	31973.893	35	0.491	0.345	0.241

DU, digital technology use; DC, digital competence; CPS, creative problem-solving.

#### 3.3.3 Hypothesis testing

To test our research hypotheses, we conducted model analyses using Mplus with TYPE = COMPLEX to account for the clustered structure of students within schools. Following best practices for complex survey data, we incorporated PISA’s final student sampling weights (W_FSTUWT) to adjust for unequal selection probabilities and ensure parameter estimates appropriately represent the target population. Our hypothesized mediation model achieved acceptable model fit [χ^2^(4) = 18.583, CFI = 0.974, TLI = 0.923, RMSEA = 0.019]. To verify the robustness of these findings, we conducted a sensitivity analysis by re-estimating the model using PISA’s standardized index of economic, social, and cultural status (ESCS) as an alternative measure of socioeconomic background. The results of this validation model remained consistent with our primary analysis. More details are provided in Supplementary Appendix A.

We first examined the direct relationships proposed in our model and reported the standardized parameter estimates. SES was positively associated with both digital use (β = 0.137, SE = 0.016, *p* < 0.001) and digital competency (β = 0.112, SE = 0.017, *p* < 0.001), thus Hypotheses 1a and 1b are supported. Additionally, digital use was positively related to digital competency (β = 0.143, SE = 0.014, *p* < 0.001), thereby supporting Hypothesis 2. In addition, digital use was positively associated with creative problem-solving (β = 0.106, SE = 0.014, *p* < 0.001), and digital competency also showed a positive association with creative problem-solving (β = 0.229, SE = 0.019, *p* < 0.001). These results provide evidence to support Hypotheses 3 and 4 in our study.

**FIGURE 1:**
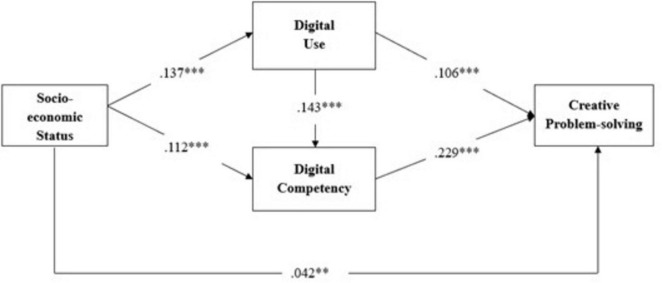
Theoretical model with parameter estimates for Study 1.

We next tested the indirect effects of SES on students’ creative problem-solving through the two mediators, digital use and digital competency. Using bias-corrected bootstrap confidence intervals with 2,000 resamples, we identified three significant mediation effects. Specifically, SES indirectly influenced creative problem-solving through digital use [indirect effect = 0.016, 95% CI (0.011, 0.022)] and through digital competency [indirect effect = 0.029, 95% CI (0.020, 0.040)]. Further, we observed a significant chain mediation effect, indicating SES influenced creative problem-solving via digital use and digital competency sequentially [indirect effect = 0.005, 95% CI (0.004, 0.007)]. Therefore, these results showed support for Hypotheses H5a, H5b, and H5c. Figure 1 shows the parameter estimates of the theoretical model.

While Study 1 provided initial evidence for the parallel mediation model using PISA data, we further conducted Study 2 to address two key objectives. First, we replicated our parallel mediation model linking SES to creative problem-solving through digital use and digital competency using primary data collection to strengthen generalizability. Second, we extended the model by incorporating the objective outcome (students’ academic ranking) to examine whether our mediation pathways predict real-world academic performance, addressing limitations of self-reported cognitive measures.

## 4 Survey study

### 4.1 Sample participants

Study 2 comprised 558 students from China, collected to provide independent replication of our PISA findings. This replication addresses growing concerns about reproducibility in educational research (Benson and Borrego, 2015) and enables verification of our findings through primary data collection using identical measurement instruments, establishing reliability and generalizability across different sampling contexts. Participants were recruited through convenience sampling from schools in a major Chinese city where the first author had established research connections.

While acknowledging the limitations of convenience sampling, we made deliberate efforts to replicate the demographic characteristics of Study 1 to ensure meaningful comparison and replication. In Study 2, gender distribution closely mirrored Study 1: 44.6% female and 55.4% male students, compared to Study 1’s 48.5% female and 51.5% male participants. Participants’ age ranged from 12 to 18 years (*M* = 15.86, SD = 0.643), spanning Grades 9–11 in high schools. Grade distribution approximated Study 1, with majority attending Grade 9 (27.8%) and Grade 10 (56.2%). Parental and student consent were obtained. Of 616 invited students, 558 submitted usable responses, yielding a 90.58% response rate.

### 4.2 Measures

To ensure comparability with Study 1, we utilized identical measurement instruments from the PISA 2022 dataset, including the same items for SES indicators, digital use, digital competency, and creative problem-solving. For the additional objective outcome in Study 2, we collected students’ academic ranking from school records based on their most recent academic performance. Students were classified into one of five ordered categories representing their rank within their grade level: top 20%, 20–40%, 40–60%, 60–80%, and bottom 20%. This variable was reverse-coded for analysis such that higher values indicate better academic standing (5 = top 20%, 1 = bottom 20%).

### 4.3 Results

#### 4.3.1 Preliminary analysis

Examination of missing data patterns revealed complete data across all variables due to the close collaboration established with participating schools, resulting in high cooperation rates among consenting participants. Our assessment of normality indicated that most study variables demonstrated acceptable levels of skewness and kurtosis. The distribution of academic ranking in our sample showed 30.1% of students in the top quintile, 21.4% in the second quintile, 27.0% in the middle quintile, 16.4% in the fourth quintile, and 5.1% in the bottom quintile, reflecting a distribution skewed toward higher academic performance that is typical in academically motivated samples.

To examine relationships among the key study variables, we conducted correlation analyses, summarized in Table 3. Digital use was positively correlated with digital competency (*r* = 0.602, *p* < 0.01) and creative problem-solving (*r* = 0.608, *p* < 0.01). In addition, digital competency demonstrated a strong positive correlation with creative problem-solving (*r* = 0.471, *p* < 0.01). Academic ranking showed significant positive correlations with digital use (*r* = 0.332, *p* < 0.01), digital competency (*r* = 0.225, *p* < 0.01), and creative problem-solving (*r* = 0.174, *p* < 0.01). For control variables, SES was significantly and positively correlated with both mother’s education (*r* = 0.345, *p* < 0.01) and father’s education (*r* = 0.331, *p* < 0.01), while mother’s and father’s education levels were strongly correlated with each other (*r* = 0.707, *p* < 0.01). Gender was negatively correlated with digital use (*r* = -0.086, *p* < 0.05) and creative problem-solving (*r* = -0.108, *p* < 0.01), and positively correlated with academic ranking (*r* = 0.184, *p* < 0.01). Cronbach’s alpha values indicated strong internal consistency for digital use (0.942), digital competency (0.960), and creative problem-solving (0.949), supporting the reliability of these measures.

**TABLE 3:** Study 2 means, standard deviations, scale reliabilities, correlations, Cronbach’s reliability in parentheses.

Variables	Mean	SD	1	2	3	4	5	6	7	8	9
1. Grade	1.024	0.164	–								
2. Gender	1.446	0.498	0.058	–							
3. Mother education	2.564	1.205	0.148^**^	0.062	–						
4. Father education	2.778	1.179	0.107^**^	0.015	0.707^**^	–					
5. SES	3.908	1.014	0.065	0.068	0.345^**^	0.331^**^	***(0.73)***				
6. Digital technology use	3.437	1.546	-0.086*	-0.045	0.038	0.024	0.139^**^	***(0.94)***			
7. Digital competency	4.955	1.143	0.014	-0.057	0.109^**^	0.065	0.148^**^	0.602^**^	***(0.96)***		
8. Creative problem-solving	4.448	1.154	-0.007	-0.108^**^	0.015	0.024	0.115^**^	0.608^**^	0.471^**^	***(0.95)***	
9. Academic ranking^a^	3.549	1.220	0.003	-0.184^**^	0.120^**^	0.102*	0.087*	0.332^**^	0.225^**^	0.174^**^	–

*N* = 585 was used for correlation analysis. M, Mean; SD, Standard Deviation. SES is the composite score of the father’s education. The mother’s education, financial evaluation, and family annual income, calculated by saving the CFA factor scores of the four measuring items. Academic ranking is the objective score of the final exam ranking of the students.

**p < 0.01; *p < 0.05.

#### 4.3.2 Construct validity

To replicate the construct validity findings from Study 1, we conducted the same series of parceled CFAs in Study 2, examining measurement model stability in a different sample. For convergent validity, we assessed individual measurement models for digital use, digital competency, and creative problem-solving. The parceled measurement model demonstrated good fit (χ = 175.939, df = 32, CFI = 0.952, TLI = 0.933, RMSEA = 0.088). All parcel loadings were substantial and statistically significant (*p* < 0.001), ranging from 0.748 to 0.995 for digital competency, .0.773 to 0.887 for digital use, and 0.901 to 0.954 for creative problem-solving. Internal consistency was excellent across all constructs, with reliability coefficients of α = 0.94 for digital use, α = 0.96 for digital competency, and α = 0.95 for creative problem-solving. The three latent factors showed moderate positive intercorrelations: digital use with digital competency (*r* = 0.582, *p* < 0.001), creative problem-solving with digital competency (*r* = 0.474, *p* < 0.001), and creative problem-solving with digital use (*r* = 0.638, *p* < 0.001).

For discriminant validity, we conducted competing model analyses comparing our hypothesized three-factor model against increasingly parsimonious alternatives (Table 4). The three-factor model (M1) distinguishing digital use, digital competency, and creative problem-solving demonstrated good fit (χ = 175.939, df = 32, CFI = 0.952, TLI = 0.933, RMSEA = 0.088). Two-factor models showed deteriorated fit: combining digital use and digital competency (M2: χ^2^ = 609.150, df = 34, CFI = 0.809, RMSEA = 0.170); combining digital use and creative problem-solving (M3: χ^2^ = 796.812, df = 34, CFI = 0.747, RMSEA = 0.196); and combining digital competency and creative problem-solving (M4: χ^2^ = 842.185, df = 34, CFI = 0.732, RMSEA = 0.202). The single-factor model (M5) demonstrated poorest fit (χ^2^ = 1361.844, df = 35, CFI = 0.560, RMSEA = 0.255). Fit indices consistently favored the three-factor model, with substantial deterioration observed as factors were combined, providing strong evidence for discriminant validity and supporting the conceptual distinctiveness of our key variables. Supplementary Appendix B provides the full measurement model results.

**TABLE 4:** Study 2 Discriminant validity for the three study variables.

Models	χ^2^	df	CFI	TLI	RMSEA
M1: default 3-factor (DU, DC, CPS)	175.939	32	0.952	0.933	0.088
M2: 2-factor (DU+DC, CPS)	609.150	34	0.809	0.748	0.170
M3: 2-factor (DU, DC+CPS)	796.812	34	0.747	0.665	0.196
M4: 2-factor (DU+CPS, DC)	842.185	34	0.732	0.646	0.202
M5: 1-factor (DU+DC+CPS)	1361.844	35	0.560	0.435	0.255

DU, digital technology use; DC, digital competence; CPS, creative problem-solving.

#### 4.3.3 Hypothesis testing

We tested our hypothesized parallel mediation model using structural equation modeling in Mplus. The model demonstrated excellent fit to the data across multiple fit indices: χ^2^ = 11.657, df = 4, CFI = 0.988, TLI = 0.948, RMSEA = 0.057. All fit indices met or exceeded conventional standards for good model fit, indicating that our theoretical model adequately represented the observed data structure and replicated the model from Study 1.

The structural path analysis revealed significant relationships that largely replicated our Study 1 findings. Supporting our first hypothesis (H1a and H1b), SES demonstrated significant positive association with both digital use (β = 0.147, SE = 0.043, *p* < 0.01) and digital competency (β = 0.073, SE = 0.034, *p* < 0.05). Consistent with our second hypothesis (H2), digital use exhibited a strong positive relationship with digital competency (β = 0.635, SE = 0.041, *p* < 0.001), replicating the relationships observed in Study 1.

For creative problem-solving, both digital use (β = 0.552, SE = 0.049, *p* < 0.001) and digital competency (β = 0.157, SE = 0.044, *p* < 0.001) showed significant positive effects, providing strong support for our third and fourth hypotheses (H3 and H4). These findings replicated the parallel mediation pathways identified in Study 1, providing support for our theoretical model across different samples and data collection approaches.

**FIGURE 2:**
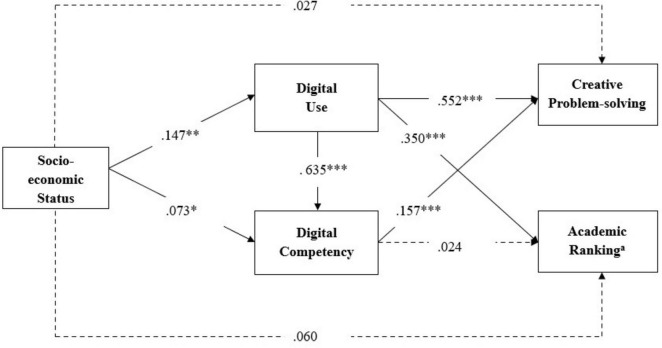
Theoretical model with parameter estimates for Study 2.

Our model extension incorporating academic ranking as an additional outcome revealed that digital use showed a significant positive effect on academic ranking of students (β = 0.350, SE = 0.064, *p* < 0.001), while digital competency did not significantly influence academic ranking (β = 0.024, SE = 0.053, *p* = 0.651). This pattern suggests that while both digital factors contribute to creative problem-solving, digital use serves as the primary pathway linking socioeconomic background to objective academic achievements. Figure 2 shows the parameter estimates of our theoretical model. We also conducted a sensitivity analysis using a probit model accounting for the ordered nature of academic ranking (Supplementary Appendix C).

Using bias-corrected bootstrap confidence intervals with 2,000 resamples, we identified four significant mediation effects that replicated the mediation results from Study 1 for Hypothesis 5. Specifically, SES indirectly influenced creative problem-solving through digital use [indirect effect = 0.081, 95% CI (0.032, 0.130)] and through digital competency [indirect effect = 0.012, 95% CI (0.001, 0.025)]. Additionally, SES indirectly influenced academic ranking through digital use [indirect effect = 0.051, 95% CI (0.019, 0.092)]. Further, we observed a significant chain mediation effect, indicating SES influenced creative problem-solving via digital use and digital competency sequentially [indirect effect = 0.015, 95% CI (0.004, 0.029)]. In contrast, the indirect effects of SES on academic ranking through digital competency [indirect effect = 0.001, 95% CI (-0.006, 0.008)] and the chain mediation path from SES to academic ranking via digital use and digital competency [indirect effect = 0.002, 95% CI (-0.007, 0.012)] were not statistically significant.

## 5 Discussion

This study establishes digital competency mechanisms as crucial mediating pathways between SES and educational outcomes. Using PISA 2022 data from Chinese students (*n* = 16,148), Study 1 identified three significant mediation pathways: SES influences creative problem-solving through digital use, through digital competency, and through the sequential pathway where digital use relates to digital competency, which then influences creative problem-solving. Study 2 provided a replication using independent primary data collection (*n* = 558) in China, confirming the robustness and generalizability of our research model across different sampling contexts. Importantly, Study 2 extended the model by incorporating the objective academic outcome (i.e., academic ranking). Beyond replicating the competency development mechanisms from Study 1, the extension showed that SES also influences students’ academic ranking through digital use.

### 5.1 Interpreting effect patterns across studies

Before discussing our theoretical and practical contributions, we address an important interpretive issue: the stronger path coefficients observed in Study 2 compared to Study 1. We contend these differences reflect genuine substantive factors in sample composition and contexts. First, Study 1 draws from PISA’s nationally representative sample across schools in various Chinese cities (including Hong Kong and Macau), capturing substantial between-school heterogeneity in resources, teaching quality, curriculum implementation, socioeconomic composition, and technological infrastructure. This heterogeneity introduces contextual variation that can attenuate individual-level relationships. For example, a student’s digital use may strongly relate to problem-solving within a school but appear weak nationally when students are embedded in vastly different educational contexts. Study 2, by contrast, samples students within a single school where structural factors are held constant. When this contextual variance is removed, individual-level relationships emerge more clearly.

Second, data collection contexts differ meaningfully. PISA students complete cognitively demanding assessments followed by extensive questionnaires in a high-stakes testing environment, potentially creating psychological distance between their performance and self-reported behaviors. Study 2 employed a focused survey where students reflected on integrated aspects of their digital learning experiences and academic outcomes within a single session, potentially enhancing their awareness of connections between digital engagement and performance. While this integrated reflection context may yield more coherent self-reports, we acknowledge that it could also introduce common method variance, which we address as a potential limitation of our study.

Third, although we used parallel measures across studies, the meaning and implementation of digital use may vary substantially across PISA’s diverse contexts spanning urban and rural schools, well-resourced and under-resourced institutions, and vastly different technological infrastructures. What constitutes “frequent digital use” or “effective digital engagement” may have different practical meanings across these varied settings. In the homogeneous context of Study 2, such “digital use” has more consistent meaning and implementation across students who share similar technology access and pedagogical experiences, potentially yielding clearer relationships with outcomes.

These considerations suggest complementary rather than contradictory evidence. Study 1 demonstrates relationships exist despite substantial contextual heterogeneity, supporting external validity. Study 2 reveals the strength of relationships in homogeneous contexts while incorporating an objective academic outcome. This convergence across methodologically distinct approaches strengthens confidence in our theoretical framework.

### 5.2 Theoretical contributions

There are three main contributions that our study aimed at contributing to existing literature. Firstly, our study attempted theoretical integration by synthesizing social cognitive theory with the digital divide framework to explain digital competency development mechanisms among students. While digital divide theory primarily operates from sociological perspectives emphasizing structural inequalities, and social cognitive theory focuses on individual learning processes, their integration provides a more comprehensive theoretical understanding of how environmental advantages may become internalized as psychological competencies, and consequently leads to better student outcomes. This synthesis moves beyond descriptive accounts of digital inequalities to examine the psychological processes through which digital competencies develop and persist across socioeconomic contexts. The progression from environmental resources to behavioral engagement to personal competencies reflects core mechanisms of observational learning and competency development, where repeated successful interactions with digital technologies build confidence and strategic thinking skills (Bandura, 1997; Schunk and DiBenedetto, 2020). Therefore, our theoretical framework attempts to explain how digital competency emerges through cycles of environmental opportunity, behavioral practice, and psychological skills development, providing a mechanistic understanding of how digital advantages may accumulate through social cognitive processes.

Building upon this theoretical foundation, our study provides an in-depth examination of sequential competency mechanisms across all three levels of the digital divide. While digital divide theory conceptually describes hierarchical levels (i.e., access, usage, and outcomes), empirical research has typically examined these levels separately or focused on direct relationships between SES and educational outcomes (van Deursen and Helsper, 2015; Scheerder et al., 2017). Our sequential mediation analyses provide robust empirical validation of this theoretical framework, revealing consistent patterns across both studies. In Study 1, we identified three significant mediation pathways: SES → digital use → creative problem-solving (indirect effect = 0.016, 95% CI [0.011, 0.022]), SES → digital competency → creative problem-solving [indirect effect = 0.029, 95% CI (0.020, 0.040)], and the complete sequential chain SES → digital use → digital competency → creative problem-solving [indirect effect = 0.005, 95% CI (0.004, 0.007)]. Study 2 replicated these findings with even stronger effect sizes, demonstrating the robustness of this sequential mechanism. Thus, these results reveal a clear developmental pathway wherein socioeconomic advantages are associated with frequent and diverse digital engagement, which subsequently relates to strategic digital competencies, ultimately corresponding to creative educational outcomes. Moreover, the modest magnitude of the SES to digital use pathway observed across both studies (β = 0.137 in Study 1, β = 0.147 in Study 2) aligns with recent longitudinal evidence demonstrating that associations between socioeconomic status and informal digital exposure are weaker than traditionally assumed (Tsang and Lo, 2025). Their findings revealed that general informal exposure (e.g., videos, print, and songs) predicted learning gains, whereas tutorial and homework time showed weak or negative associations. This pattern suggests that readily available low-cost digital platforms may reduce resource-based barriers to basic access. While Study 1 shows relatively comparable path coefficients across the sequential chain, Study 2 demonstrates substantially stronger effects from digital use to outcomes (β = 0.552 to creative problem-solving, β = 0.350 to academic ranking) compared to the initial SES to digital use association. This cross-study pattern suggests that while digital access has become somewhat democratized through free platforms, transforming this access into meaningful educational benefits through sustained engagement and competency development represents the critical challenge for educational equity. Such cross-validation demonstrates how environmental resources progress through behavioral engagement to internalized personal competencies, filling a significant theoretical gap in understanding the psychological mechanisms underlying digital educational advantages. The sequential nature of this process indicates that digital competencies are not simply distributed based on access availability, but develop through sustained engagement patterns that are themselves influenced by socioeconomic factors and guided by social cognitive principles. These finding advance theoretical understanding beyond static descriptions of digital divides toward a more dynamic developmental models of how the differential influences of digital access, usage patterns, and competency development on educational outcomes demonstrate reciprocal determinism, where environmental factors, behavioral patterns, and personal skills interact dynamically to influence learning trajectories (Zimmerman and Schunk, 2011).

Finally, our research provides more theoretical precision by differentiating the roles of procedural digital use and strategic digital competency in predicting distinct educational outcomes. Our findings provide empirical evidence for this theoretical distinction, particularly through the extension of Study 2 by incorporating both creative problem-solving and academic ranking outcomes. While digital use significantly predicted both creative problem-solving (β = 0.552, *p* < 0.001) and academic ranking (β = 0.350, *p* < 0.001), digital competency selectively influenced only creative problem-solving (β = 0.157, *p* < 0.001) with no significant association with academic ranking (β = 0.024, *p* = 0.651). This differential pattern provides preliminary evidence for distinct cognitive mechanisms. Digital use, representing procedural engagement and frequent exposure to digital environments, may facilitate learning through cognitive load reduction and familiarity effects. This form of engagement likely reduces cognitive demands related to interface navigation and routine technological tasks, thereby freeing cognitive resources for efficient processing of educational content (Sweller et al., 2019; van Merriënboer and Sweller, 2010). Students engaging frequently with digital learning environments gain repeated exposure to structured information, directly enhancing conventional academic performance through practice effects and procedural fluency (Fraillon et al., 2020). Conversely, digital competency captures higher-order strategic skills involving information synthesis, critical evaluation, and adaptive application of digital resources. These competencies align with complex cognitive processes requiring ideational fluency, flexible thinking, and innovative problem-solving approaches (Mishra and Koehler, 2006; Voogt and Roblin, 2012). Digital competency strongly corresponds with creative educational outcomes that demand originality and adaptability, but does not necessarily translate into traditional academic metrics emphasizing memorization and standardized procedures (Runco and Acar, 2012; Beghetto and Kaufman, 2014). This theoretical distinction aligns with multiliteracies perspectives on digital learning (Lo, 2020). From this perspective, digital learning spaces (including social platforms, collaborative environments, and multimodal composition tools) create affordances for observational learning, guided practice, and multimodal authorship that cultivate strategic competencies such as curation, collaboration, and metacognitive regulation. These multiliteracy competencies map naturally onto creative problem-solving tasks requiring flexibility and innovation, yet conventional academic ranking systems may not capture them, instead emphasizing routinized curricular knowledge. Computer-mediated discourse and multimodal composition cultivate strategic thinking skills that align with open-ended creative challenges, while traditional assessments prioritize standardized procedures and memorization that may be facilitated more directly through procedural digital familiarity. This distinction provides a mechanistic understanding of why different forms of digital engagement produce varying educational benefits, challenging assumptions of uniform technological effects and providing precision for future theoretical developments in digital learning research (Organisation for Economic Co-operation Development [OECD], 2020; Warschauer, 2011).

### 5.3 Practical implications

Our findings provide concrete guidance for educational policies and practices by demonstrating the sequential pathways through which socioeconomic advantages translate into educational outcomes via digital engagement. The empirical validation of social cognitive mechanisms in digital competency reveals that such development follows a trajectory where procedural engagement serves as a prerequisite for strategic mastery (Bandura, 2006; van Deursen and Helsper, 2015). Educational interventions must therefore address both stages of this progression, moving beyond hardware provision toward sustained and guided engagement opportunities (Warschauer, 2011; Livingstone and Helsper, 2007). Suggested by Lo (2020), effective interventions should create digital learning spaces that afford observational learning, collaborative authorship, and multimodal production. Such learning environments cultivate the strategic competencies identified in our dual-pathway framework. Specific intervention designs might include: (a) learning management system projects requiring resource curation and multimodal composition to build synthesis and evaluation skills; (b) telecollaborative exchanges scaffolding intercultural problem-solving and ideational fluency through structured digital dialog; and (c) game-based activities developing metacognitive regulation and flexible thinking. School initiatives should bundle device access with explicit instruction in both procedural fluency (navigating platforms, basic operations) and strategic multiliteracy skills (synthesizing information, evaluating content, creating multimodal artifacts). Such comprehensive approaches require providing scaffolding and guidance to mitigate potential risks including distraction or superficial engagement, while ensuring that disadvantaged students receive additional technical support and extended supervised practice opportunities to develop both foundational and advanced competencies (Hargittai and Hinnant, 2008).

Realizing these intervention designs requires substantial investment in teacher preparation and professional development. Teachers need explicit training in recognizing how socioeconomic factors shape students’ digital engagement trajectories and competency development pathways (Bourdieu and Passeron, 1990; Gonzales et al., 2020). Professional development programs should equip educators to implement multiliteracy pedagogies that leverage digital tools for collaborative knowledge construction rather than treating technology as merely a content delivery mechanism (Lo, 2020). This includes developing teachers’ capacity to design learning experiences that support reciprocal determinism through coordinating environmental resources, facilitating meaningful behavioral engagement, and building students’ personal competencies through structured observational learning and self-efficacy development (Bandura, 1997; Zimmerman and Schunk, 2011). Teacher preparation must also address the tendency to assume digital competencies develop naturally through exposure, ensuring educators understand that strategic skills require explicit instruction and guided practice regardless of students’ apparent technological fluency (Scheerder et al., 2017; DiMaggio and Garip, 2012). Additionally, schools need organizational structures supporting sustained technology integration, including dedicated instructional design support, protected planning time for developing digital learning activities, and professional learning communities where teachers can collaboratively refine their multiliteracy pedagogical approaches.

Assessment systems should incorporate measures of both digital use patterns and strategic digital competencies to provide comprehensive monitoring of students’ progress along the full developmental pathway (Fraillon et al., 2020). Traditional focus on academic achievement alone may miss critical competency gaps that limit creative problem-solving development, particularly among socioeconomically disadvantaged students who may demonstrate adequate procedural skills but lack opportunities to develop strategic skills (van Deursen and Helsper, 2015). Schools should implement assessment frameworks that capture both types of outcomes, recognizing that students may demonstrate different patterns of digital competency development depending on their engagement opportunities and instructional experiences (Schunk and DiBenedetto, 2020). This nuanced understanding can inform more targeted and effective interventions that address the specific competency gaps most relevant to desired educational outcomes, ensuring that technology policies do not celebrate superficial access gains while leaving deeper developmental divides untouched (Hargittai and Hinnant, 2008; Livingstone and Helsper, 2007).

### 5.4 Limitations and future research directions

Our research presents several limitations that warrant attention and indicate directions for future investigation. An important limitation concerns the aggregation of qualitatively different activities within our digital use construct. Our measure aggregated school-directed academic engagement with learner-initiated informal activities, yet emerging research suggests these activity types may operate through distinct mechanisms with differential developmental effects (Tsang and Lo, 2025). This aggregation may obscure theoretically meaningful distinctions that could refine understanding of how specific forms of digital engagement translate into competency development. Future research should employ typology-sensitive measures that separately assess: (a) formal academic activities (e.g., learning management systems, online coursework, tutorial platforms), and (b) informal engagement (e.g., recreational reading, social communication, gaming). Self-report measures could be triangulated with objective indicators such as device usage logs or structured parent-student co-reports to enhance measurement validity (Tsang and Lo, 2025). Such disaggregation would enable more precise tests of our dual-pathway framework and provide actionable guidance for educational interventions by identifying which forms of digital engagement merit targeted cultivation.

Another limitation of our research is the reliance on self-reported measures for digital use, digital competency, and creative problem-solving constructs. While Study 1 utilized exclusively self-report scales from the PISA dataset, Study 2 partially addressed this concern by incorporating academic ranking as an objective outcome measure alongside the self-reported variables. Nevertheless, the core predictor and mediator variables remain based on self-assessments, which may be subject to social desirability responding (King and Bruner, 2000) or limited metacognitive accuracy among adolescent participants (Aesaert et al., 2017). This could potentially inflate relationships among self-reported constructs due to shared method variance (Podsakoff et al., 2003). Specifically, students with high general self-efficacy may consistently overestimate both their usage frequency and their competency levels, artificially strengthening the observed associations between these variables. We conducted a common latent factor analysis for study 2 (see discussions in Supplementary Appendix D). Future research would benefit from incorporating performance-based assessments of digital competency and objective measures of digital use patterns to further validate these self-report measures. Such objective metrics are essential to disentangle actual technical proficiency from confidence-based reporting, ensuring that the identified developmental pathways reflect genuine skill acquisition rather than self-perception biases.

In addition, although our theoretical framework implies a developmental sequence where socioeconomic factors influence digital behaviors which in turn foster competencies, our findings rely primarily on cross-sectional data. This limits our ability to infer causal relationships among SES, digital use, digital competency, and academic outcomes. Therefore, the directed pathways identified in our structural models should be interpreted as associations consistent with our proposed theoretical mechanisms rather than definitive causal effects. To strengthen causal interpretations, future studies could employ longitudinal designs or experimental interventions to examine how digital competencies develop and influence outcomes over time. For instance, longitudinal designs with multiple measurement waves would enable researchers to establish temporal precedence among variables and test causal sequences through cross-lagged panel models (Maxwell and Cole, 2007; Little, 2013).

Finally, our samples were drawn exclusively from Chinese educational contexts characterized by a highly competitive, exam-oriented culture. This cultural specificity might constrain the generalizability of our findings to other educational environments, especially those that prioritize different learning goals and pedagogical approaches. Future research should replicate and test our model in diverse contexts, including Western educational systems, rural or economically disadvantaged regions, and countries with varying educational policies, to confirm the robustness and cultural applicability of our theoretical framework.

## 6 Conclusion

Our study advances understanding of digital competency development by demonstrating how socioeconomic advantages translate into educational outcomes through sequential psychological pathways. Using two complementary studies, we empirically validated the mechanisms through which SES is associated with digital use, which subsequently develops digital competency and ultimately influences creative problem-solving. Our theoretical integration of social cognitive theory with digital divide frameworks provides a mechanistic understanding of competency development processes, while our empirical differentiation between procedural digital use and strategic digital competency reveals their distinct educational functions. These findings offer evidence-based guidance for educational interventions that target sequential competency development rather than merely providing technology access, emphasizing sustained engagement opportunities and strategic skill cultivation. Future studies should explore how these sequential pathways operate across diverse cultural contexts and examine longitudinal competency development trajectories to further refine intervention timing and design.

## Data Availability

The raw data supporting the conclusions of this article will be made available by the authors, without undue reservation.

## References

[B1] Abedini A. Abedin B. Zowghi D. (2024). A framework of environmental, personal, and behavioral factors of adult learning in online communities of practice. *Inf. Syst. Front.* 26 1201–1218. 10.1007/s10796-023-10417-2

[B2] Adeoye M. A. Jimoh H. (2023). Problem-solving skills among 21st-century learners toward creativity and innovation ideas. *Think. Skills Creat.* 6:1. 10.23887/tscj.v6i1.62708

[B3] Aesaert K. Voogt J. Kuiper E. van Braak J. (2017). Accuracy and bias of ICT self-efficacy: An empirical study into students’ over-and underestimation of their ICT competences. *Comput. Hum. Behav.* 75, 92–102. 10.1016/j.chb.2017.05.010

[B4] Afzal A. Khan S. Daud S. Ahmad Z. Butt A. (2023). Addressing the digital divide: Access and use of technology in education. *J. Soc. Sci. Rev.* 3 883–895. 10.54183/jssr.v3i2.326

[B5] Bandura A. (1986). *Social foundations of thought and action: A social cognitive theory.* Englewood Cliffs, NJ: Prentice-Hall.

[B6] Bandura A. (1997). *Self-efficacy: The exercise of control.* New York, NY: Freeman.

[B7] Bandura A. (2006). “Guide for constructing self-efficacy scales,” in *Self-efficacy beliefs of adolescents*, eds. Urdan T. Pajares F. (Greenwich, CT: Information Age Publishing), 5, 307–337.

[B8] Barana A. Marchisio M. Sacchet M. (2021). Interactive feedback for learning mathematics in a digital learning environment. *Educ. Sci.* 11:279. 10.3390/educsci11060279

[B9] Beghetto R. A. Kaufman J. C. (2014). Classroom contexts for creativity. *High Abil. Stud.* 25 53–69. 10.1080/13598139.2014.905247

[B10] Benson L. Borrego M. (2015). The role of replication in engineering education research. *J. Eng. Educ.* 104 388–392. 10.1002/jee.20082

[B11] Blanc S. Conchado A. Benlloch-Dualde J. V. Monteiro A. Grindei L. (2025). Digital competence development in schools: A study on the association of problem-solving with autonomy and digital attitudes. *Int. J. STEM Educ.* 12:13. 10.1186/s40594-025-00534-6

[B12] Bourdieu P. Passeron J. C. (1990). *Reproduction in education, society and culture*, 2nd Edn. London: Sage Publications.

[B13] Cha W. Hong M. Glassman M. Anderman E. M. Lin T. J. (2025). The roles of technology efficacy and networking agency in elementary students’ engagement in online and face-to-face technology-mediated learning. *Br. J. Educ. Technol.* 56 2623–2646. 10.1111/bjet.13597

[B14] Cheung A. C. Slavin R. E. (2013). The effectiveness of educational technology applications for enhancing mathematics achievement in K-12 classrooms: A meta-analysis. *Educ. Res. Rev.* 9, 88–113.

[B15] Chmielewski A. K. (2019). The global increase in the socioeconomic achievement gap, 1964 to 2015. *Am. Sociol. Rev.* 84 517–544. 10.1177/0003122419847165

[B16] Conneely C. Lawlor J. Tangney B. (2020). Developing critical thinking, collective creativity skills and problem solving through playful design jams. *Think. Skills Creat.* 37:100696. 10.1016/j.tsc.2020.100696

[B17] DiMaggio P. Garip F. (2012). Network effects and social inequality. *Annu. Rev. Sociol.* 38 93–118. 10.1146/annurev.soc.012809.102545

[B18] DiStefano C. Zhu M. Mindrila D. (2009). Understanding and using factor scores: Considerations for the applied researcher. *Pract. Assess. Res. Eval.* 14:1. 10.7275/da8t-4g52

[B19] Duncan G. J. Magnuson K. Votruba-Drzal E. (2017). Moving beyond correlations in assessing the consequences of poverty. *Annu. Rev. Psychol.* 68 413–434. 10.1146/annurev-psych-010416-044224 27648987 PMC6108837

[B20] Enders C. K. (2001). The performance of the full information maximum likelihood estimator in multiple regression models with missing data. *Educ. Psychol. Meas.* 61 713–740. 10.1177/00131640121971482

[B21] Fraillon J. Ainley J. Schulz W. Friedman T. Duckworth D. (2020). *Preparing for life in a digital world: IEA international computer and information literacy study 2018 international report.* Cham: Springer. 10.1007/978-3-030-38781-5

[B22] Getenet S. Cantle R. Redmond P. Albion P. (2024). Students’ digital technology attitude, literacy and self-efficacy and their effect on online learning engagement. *Int. J. Educ. Technol. High. Educ.* 21:3. 10.1186/s41239-023-00437-y

[B23] Godsk M. Møller K. L. (2025). Engaging students in higher education with educational technology. *Educ. Inf. Technol.* 30 2941–2976. 10.1007/s10639-024-12901-x

[B24] Goldhammer F. Gniewosz G. Zylka J. (2016). “ICT engagement in learning environments,” in *Assessing contexts of learning: An international perspective*, eds Kuger S. Klieme E. Jude N. Kaplan Eds D. (Cham: Springer International Publishing), 331–351.

[B25] Gonzales A. L. Calarco J. M. Lynch T. (2020). Technology problems and student achievement gaps: A validation and extension of the technology maintenance construct. *Commun. Res.* 47 750–770. 10.1177/0093650218796366

[B26] Gosselin D. Cooper S. Bonnstetter R. J. Bonnstetter B. J. (2013). Exploring the assessment of twenty-first century professional competencies of undergraduate students in environmental studies through a business—academic partnership. *J. Environ. Stud. Sci.* 3 359–368. 10.1007/s13412-013-0140-1

[B27] Haelermans C. Huijgen T. Jacobs M. Levels M. van der Velden R. van Vugt L. (2020). Using data to advance educational research, policy, and practice: Design, content, and research potential of the Netherlands Cohort Study on Education. *Eur. Sociol. Rev.* 36 643–662. 10.1093/esr/jcaa027 40318190

[B28] Hammer M. Scheiter K. Stürmer K. (2021). New technology, new role of parents: How parents’ beliefs and behavior affect students’ digital media self-efficacy. *Comput. Hum. Behav.* 116:106642. 10.1016/j.chb.2020.106642

[B29] Hargittai E. Hinnant A. (2008). Digital inequality: Differences in young adults’ use of the Internet. *Commun. Res.* 35 602–621. 10.1177/0093650208321782

[B30] Hashim M. M. Tlemsani I. Matthews R. (2022). Higher education strategy in digital transformation. *Educ. Inf. Technol.* 27 3171–3195. 10.1007/s10639-021-10739-1 34539217 PMC8438547

[B31] Hu L. T. Bentler P. M. (1999). Cutoff criteria for fit indexes in covariance structure analysis: Conventional criteria versus new alternatives. *Struct. Equ. Model.* 6 1–55. 10.1080/10705519909540118

[B32] Karrer A. Klemke R. Specht M. (2023). *10 ways artificial intelligence is transforming instructional design.* Boulder: CO: Educause Review.

[B33] King M. F. Bruner G. C. (2000). Social desirability bias: A neglected aspect of validity testing. *Psychol. Mark.* 17 79–103. 10.1002/(SICI)1520-6793(200002)17:2<79::AID-MAR2>3.0.CO;2-0

[B34] Kong S. C. Wang Y. Q. (2023). Monitoring cognitive development through the assessment of computational thinking practices: A longitudinal intervention on primary school students. *Comput. Hum. Behav.* 145:107749. 10.1016/j.chb.2023.107749

[B35] Kong S. C. Wang Y. Q. (2024). Dynamic interplays between self-regulated learning and computational thinking in primary school students through animations and worksheets. *Comput. Educ.* 220:105126. 10.1016/j.compedu.2024.105126

[B36] Kong S. C. Wang Y. Q. Lai M. (2019). “Development and validation of an instrument for measuring digital empowerment of primary school students,” in *Proceedings of the ACM conference on global computing education*, (New York, NY: Association for Computing Machinery, Inc), 172–177.

[B37] Kuo-Hsun J. (2021). The digital divide at school and at home: A comparison between schools by socioeconomic level across 47 countries. *Int. J. Comp. Sociol.* 62 115–140. 10.1177/00207152211023540

[B38] Lewis S. (2014). The OECD, PISA and educational governance: A call to critical engagement. *Discourse* 35 317–327. 10.1080/01596306.2014.899833

[B39] Li F. Cheng L. Wang X. Shen L. Ma Y. Islam A. Y. M. (2025). The causal relationship between digital literacy and students’ academic achievement: A meta-analysis. *Humanit. Soc. Sci. Commun.* 12 1–12. 10.1057/s41599-025-04399-6

[B40] Li J. Xue E. (2023). Dynamic interaction between student learning behaviour and learning environment: Meta-analysis of student engagement and its influencing factors. *Behav. Sci.* 13:59. 10.3390/bs13010059 36661631 PMC9855184

[B41] Li Z. Oon P. T. (2024). The transfer effect of computational thinking (CT)-STEM: A systematic literature review and meta-analysis. *Int. J. STEM Educ.* 11:44. 10.1186/s40594-024-00498-z

[B42] Little T. D. (2013). *Longitudinal structural equation modeling.* New York, NY: Guilford Press.

[B43] Little T. D. Cunningham W. A. Shahar G. Widaman K. F. (2002). To parcel or not to parcel: Exploring the question, weighing the merits. *Struct. Equ. Model.* 9 151–173. 10.1207/S15328007SEM0902_1

[B44] Little T. D. Rhemtulla M. Gibson K. Schoemann A. M. (2013). Why the items versus parcels controversy needn’t be one. *Psychol. Methods* 18 285–300. 10.1037/a0033266 23834418 PMC3909043

[B45] Livingstone S. Helsper E. J. (2007). Gradations in digital inclusion: Children, young people, and the digital divide. *New Media Soc.* 9 671–696. 10.1177/1461444807080335

[B46] Livingstone S. Lemish D. Lim S. S. Bulger M. Cabello P. Claro M. (2017). Global perspectives on children’s digital opportunities: An emerging research and policy agenda. *Pediatrics* 140 S137–S141. 10.1542/peds.2016-1758S 29093049

[B47] Lo N. P. K. (2020). “Revolutionising language teaching and learning via digital media innovations,” in *Learning environment and design. educational communications and technology yearbook*, eds Ma W. W. Tong K. W. Tso W. B. A. (Singapore: Springer), 10.1007/978-981-15-8167-0_15

[B48] Lopez-Garrido G. (2023). *Bandura’s self-efficacy theory of motivation in psychology.* London: Simply Psychology.

[B49] Ma H. Ismail L. (2025). Bibliometric analysis and systematic review of digital competence in education. *Humanit. Soc. Sci. Commun.* 12:185. 10.1057/s41599-025-04401-1

[B50] Maxwell S. E. Cole D. A. (2007). Bias in cross-sectional analyses of longitudinal mediation. *Psychol. Methods* 12 23–44. 10.1037/1082-989X.12.1.23 17402810

[B51] Merchant G. (2013). *Virtual literacies: Interactive spaces for children and young people.* London: Routledge, 10.4324/9780203079818

[B52] Mishra P. Koehler M. J. (2006). Technological pedagogical content knowledge: A framework for teacher knowledge. *Teach. Coll. Rec.* 108 1017–1054. 10.1111/j.1467-9620.2006.00684.x

[B53] Muthén L. K. Muthén B. O. (2017). *Mplus user’s guide*, 8th Edn. Los Angeles, CA: Muthén & Muthén.

[B54] Njeri M. Taym A. (2024). Analysing the power of socioeconomic status on access to technology-enhanced learning in secondary schools. *Res. Stud. Engl. Lang. Teach. Learn.* 2 223–250. 10.62583/rseltl.v2i4.55

[B55] Organisation for Economic Co-operation Development [OECD] (2020). *PISA 2018 results (Volume V): Effective policies, successful schools.* Paris: OECD Publishing, 10.1787/ca768d40-en

[B56] Organisation for Economic Co-operation Development [OECD] (2025). *PISA 2025 learning in the digital world framework.* Paris: OECD Publishing.

[B57] Pifarré M. Vendrell M. (2022). The role of digital technologies to promote collaborative creativity in language education. *Front. Psychol.* 13:828981. 10.3389/fpsyg.2022.828981 35222209 PMC8865196

[B58] Podsakoff P. M. MacKenzie S. B. Lee J. Y. Podsakoff N. P. (2003). Common method biases in behavioral research: A critical review of the literature and recommended remedies. *J. Appl. Psychol.* 88 879–903. 10.1037/0021-9010.88.5.879 14516251

[B59] Raihan M. M. Subroto S. Chowdhury N. Koch K. Ruttan E. Turin T. C. (2025). Dimensions and barriers for digital (in) equity and digital divide: A systematic integrative review. *Digit. Transform. Soc.* 4 111–127. 10.1108/DTS-04-2024-0054

[B60] Rideout V. Katz V. S. (2016). *Opportunity for all? Technology and learning in lower-income families.* New York, NY: Joan Ganz Cooney Center at Sesame Workshop.

[B61] Rosales-Márquez C. Carbonell-García C. E. Miranda-Vargas V. Diaz-Zavala R. Laura-De La Cruz K. M. (2025). Self-confidence as a predictor of digital skills: A fundamental pillar for the digitalization of higher education. *Front. Educ.* 9:1515033. 10.3389/feduc.2024.1515033

[B62] Runco M. A. Acar S. (2012). Divergent thinking as an indicator of creative potential. *Creat. Res. J.* 24 66–75. 10.1080/10400419.2012.652929

[B63] Scheerder A. Van Deursen A. Van Dijk J. (2017). Determinants of Internet skills, uses and outcomes: A systematic review of the second- and third-level digital divide. *Telemat. Inform.* 34 1607–1624. 10.1016/j.tele.2017.07.007

[B64] Schunk D. H. DiBenedetto M. K. (2020). Motivation and social cognitive theory. *Contemp. Educ. Psychol.* 60:101832. 10.1016/j.cedpsych.2019.101832

[B65] Schunk D. H. Usher E. L. (2019). “Social cognitive theory and motivation,” in *The Oxford handbook of human motivation*, 2nd Edn, ed. Ryan R. M. (Oxford: Oxford University Press), 11–26.

[B66] Song D. Bonk C. J. (2016). Motivational factors in self-directed informal learning from online learning resources. *Cogent Educ.* 3:1205838. 10.1080/2331186X.2016.1205838

[B67] Sweller J. van Merriënboer J. J. G. Paas F. (2019). Cognitive architecture and instructional design: Twenty years later. *Educ. Psychol. Rev.* 31 261–292. 10.1007/s10648-019-09465-5

[B68] Tondeur J. Sinnaeve I. Houtte M. Braak J. (2010). Ict as cultural capital: The relationship between socioeconomic status and the computer-use profile of young people. *New Media Soc.* 13 151–168. 10.1177/1461444810369245

[B69] Tsang A. Lo N. (2025). A study of FL children’s exposure, family backgrounds, and vocabulary learning. *Appl. Ling. Rev.* 10.1515/applirev-2024-0244 [Epub ahead of print].

[B70] Tzafilkou K. Perifanou M. Economides A. A. (2022). Development and validation of students’ digital competence scale (SDiCoS). *Int. J. Educ. Technol. High. Educ.* 19:30. 10.1186/s41239-022-00330-0 35602658 PMC9107949

[B71] Ulfert-Blank A. S. Schmidt I. (2022). Assessing digital self-efficacy: Review and scale development. *Comput. Educ.* 191:104626. 10.1016/j.compedu.2022.104626

[B72] van Deursen A. J. Helsper E. J. (2015). The third-level digital divide: Who benefits most from being online? *Commun. Inf. Technol. Annu.* 10 29–52. 10.1108/S2050-206020150000010002

[B73] van Deursen A. J. van Dijk J. A. (2019). The first-level digital divide shifts from inequalities in physical access to inequalities in material access. *New Media Soc.* 21 354–375. 10.1177/1461444818797082 30886536 PMC6380454

[B74] van Merriënboer J. J. Sweller J. (2010). Cognitive load theory in health professional education: Design principles and strategies. *Med. Educ.* 44 85–93. 10.1111/j.1365-2923.2009.03498.x 20078759

[B75] Voogt J. Roblin N. P. (2012). A comparative analysis of international frameworks for 21st century competences: Implications for national curriculum policies. *J. Curric. Stud.* 44 299–321. 10.1080/00220272.2012.668938

[B76] Wang X. Wang Z. Wang Q. Chen W. Pi Z. (2021). Supporting digitally enhanced learning through measurement in higher education: Development and validation of a university students’ digital competence scale. *J. Comput. Assist. Learn.* 37 1063–1076. 10.1111/jcal.12546

[B77] Warschauer M. (2011). *Learning in the cloud: How (and why) to transform schools with digital media.* New York, NY: Teachers College Press.

[B78] Zimmerman B. J. Schunk D. H. (2011). “Self-regulated learning and performance: An introduction and an overview,” in *Handbook of self-regulation of learning and performance*, eds Schunk D. H. Zimmerman B. J. (Milton Park: Routledge).

