# Supplementary material for: Socioeconomic status and educational inequality: digital competency pathways to creative problem-solving and academic performance
Source: Front Psychol. 2026 Jan 15;16:1690989. doi: 10.3389/fpsyg.2025.1690989 (PMC12852326; doi:10.3389/fpsyg.2025.1690989)
Supplement: Supplementary file 1 [file Supplementary_file_1.docx]

**Appendix A**

**Robustness Check Comparing Custom SES with PISA ESCS**

To maintain measurement consistency across Study 1 (PISA data) and Study 2 (school survey), we constructed a simplified SES composite from four indicators: mother’s education, father’s education, family financial evaluation, and annual family income. PISA’s Economic, Social and Cultural Status (ESCS) index is more comprehensive, incorporating parents’ occupation and an extensive home possessions inventory (books, cultural items, educational resources, technology access). We conducted this robustness analysis to assess whether our substantive conclusions hold when using PISA’s standardized ESCS measure.

**Correlation Between Measures**

Our custom SES factor correlates substantially with PISA ESCS (r = .693, p < .001), indicating considerable construct overlap (48% shared variance) while capturing some unique aspects of socioeconomic status.

**Model Comparison**

Model fit was comparable across specifications (Custom SES: χ²(4) = 18.583, CFI = .974, TLI = .923, RMSEA = .019; ESCS: χ²(4) = 18.101, CFI = .983, TLI = .950, RMSEA = .019).

**
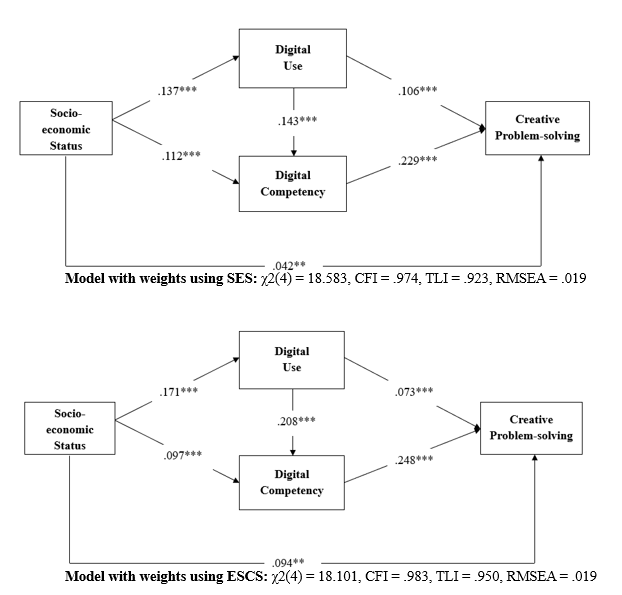
**

**Path Coefficient and Indirect Effects Comparison**

| **Structural Path** | **Custom**  **SES** | **PISA**  **ESCS** | **Difference** |
| --- | --- | --- | --- |
| ***Direct Effects*** |  |  |  |
| SES → Digital Use | .137*** | .171*** | +.034 |
| SES → Digital Competency | .112*** | .097*** | –.015 |
| Digital Use → Digital Competency | .143*** | .208*** | +.065 |
| Digital Use → Creative Problem-Solving | .106*** | .073*** | –.033 |
| Digital Competency → Creative Problem-Solving | .229*** | .248*** | +.019 |
| SES → Creative Problem-Solving (direct) | .042** | .094** | +.052 |
| ***Indirect Effects (Mediation Pathways)*** |  |  |  |
| SES → Digital Use → Creative Problem-Solving | .015*** | .012** | –.003 |
| SES → Digital Competency → Creative Problem-Solving | .026*** | .024*** | –.002 |
| SES → Digital Use → Digital Competency → Creative PS | .004** | .007*** | +.003 |
| Total Indirect Effect | .045*** | .043*** | –.002 |

***Note.*** Standardized coefficients from weighted structural equation models. Both models control for student grade and gender. **p < .01. ***p < .001.

**Conclusion**

While specific path magnitudes vary depending on how comprehensively SES is measured, our core theoretical conclusions remain robust. We retained the custom SES measure in main analyses to ensure cross-study measurement consistency, as Study 2 data collection could not replicate ESCS’s comprehensive home inventory.

**Appendix B**

**Item-Level Measurement Properties for Non-parceled Confirmatory Factor Analyses**

This supplement provides detailed item-level measurement properties for all multi-item constructs across both studies. While our main analyses employed item parceling to reduce model complexity and improve estimation stability, we present non-parceled measurement models here to demonstrate that parceling did not mask measurement problems and that all constructs exhibit strong psychometric properties at both item and parcel levels.

**Study 1: Internal Consistency and Validity Statistics**

| **Construct** | **Loading Range** | **ω** | **AVE** | **Scale Type** |
| --- | --- | --- | --- | --- |
| Digital Use (DU) | .441 – .793 | .88 | .51 | PISA Scale |
| Digital Competency (DC) | .520 – .848 | .94 | .54 | PISA Scale |
| Creative Problem-Solving (CPS) | .638 – .876 | .93 | .59 | PISA Scale |

**Study 2: Internal Consistency and Validity Statistics**

| **Construct** | **Loading Range** | **ω** | **AVE** | **Scale Type** |
| --- | --- | --- | --- | --- |
| Digital Use (DU) | .788 – .926 | .95 | .75 | 7-point Likert |
| Digital Competency (DC) | .639 – .911 | .96 | .62 | 7-point Likert |
| Creative Problem-Solving (CPS) | .743 – .921 | .96 | .71 | 7-point Likert |

**Model Fit: Parceled vs. Non-parceled Models**

| **Model** | **χ2 (df)** | **CFI** | **TLI** | **RMSEA** |
| --- | --- | --- | --- | --- |
| **Study 1** |  |  |  |  |
| Parceled (Main study) | 3108.584 (32) | .951 | .931 | .078 |
| Non-parceled | 12388.257 (423) | .925 | .917 | .042 |
| **Study 2** |  |  |  |  |
| Parceled (Main study) | 175.939 (32) | .952 | .933 | .088 |
| Non-parceled | 1332.045 (423) | .925 | .917 | .061 |

**Cross-Study of Item-Level Psychometric Properties**

| **Construct** | **Study 1** | **Study 2** | **Difference** |
| --- | --- | --- | --- |
| **Digital Use (DU)** |  |  |  |
| Loading range | .441 – .793 | .788 – .926 |  |
| **ω** | .88 | .95 | +.07 |
| AVE | .51 | .75 | +.24 |
| **Digital Competency (DC)** |  |  |  |
| Loading range | .520 – .848 | .639 – .911 |  |
| **ω** | .94 | .96 | +.02 |
| AVE | .54 | .62 | +.08 |
| **Creative Problem-Solving (CPS)** |  |  |  |
| Loading range | .638 – .876 | .743 – .921 |  |
| **ω** | .93 | .96 | +.03 |
| AVE | .59 | .71 | +.12 |

**Cross-Study of Discriminant Validity Assessment**

| **Study 1** | **DU** | **DC** | **CPS** |
| --- | --- | --- | --- |
| **Digital Use (DU)** | **.51** |  |  |
| **Digital Competency (DC)** | .219 (.048) | **.54** |  |
| **Creative Problem-Solving (CPS)** | .140 (.020) | .303 (.092) | **.59** |
| **Study 2** |  |  |  |
| **Digital Use (DU)** | **.75** |  |  |
| **Digital Competency (DC)** | .115** (.013) | **.62** |  |
| **Creative Problem-Solving (CPS)** | .011 (.000) | .482*** (.232) | **.71** |

***Note.*** Diagonal elements (bold) represent the Average Variance Extracted (AVE). Off-diagonal elements represent the standardized correlation between factors, with squared correlations in parentheses. Discriminant validity is supported as all AVE values exceed the shared variance (squared correlations) between constructs. *** p < .001, ** p < .01.

**Appendix C**

**Comparison of Structural Equation Models Treating Academic Ranking as Continuous vs. Ordinal**

To ensure the robustness of our findings regarding the academic ranking outcome, we conducted a sensitivity analysis comparing our primary model specification (treating the 5-point ranking variable as continuous using the MLR estimator) against an alternative specification treating the ranking as categorical (ordinal).

**Comparison of Standardized Path Coefficients and Model Fit Indices**

| **Structural Path** | **Primary Model (Continuous)** | **Sensitivity Model (Ordinal)** | **Difference** |
| --- | --- | --- | --- |
| **Direct Effects** |  |  |  |
| SES 🡪 Digital Use | .147** | .156** | +.009 |
| SES 🡪 Digital Competency | .073* | .071* | -.002 |
| SES 🡪 Creative Problem-solving | .027 | .027 | .000 |
| SES 🡪 Academic Ranking | .060 | .058 | -.002 |
|  |  |  |  |
| Digital Use 🡪 Digital Competency | .635*** | .639*** | +.004 |
| Digital Use 🡪 Creative Problem-solving | .552*** | .552*** | .000 |
| Digital Use 🡪Academic Ranking | .350* | .316* | -.034 |
|  |  |  |  |
| Digital Competency 🡪 Creative Problem-solving | .157*** | .157*** | .000 |
| Digital Competency 🡪 Academic Ranking | .024 | .018 | -.006 |
|  |  |  |  |
| **Model Fit Indices** |  |  |  |
| Chi-Square | 11.657 (df=4) | 11.015 (df=4) |  |
| CFI | .988 | .986 |  |
| TLI | .948 | .936 |  |
| RMSEA | .057 | .055 |  |

**Appendix D**

**Common Latent Factor (CLF) Test for Study 2**

To address the potential for common method bias in Study 2 (where all predictors and the creative problem-solving outcome were self-reported), we conducted a Common Latent Factor (CLF) analysis following the guidelines of Williams et al. (2010). We estimated a model in which all manifest variables (parcels) loaded on their theoretical constructs (digital competency, digital use, creative problem-solving) and simultaneously loaded on a single, uncorrelated method factor.

We compared the model fit and standardized factor loadings of the theoretical constructs in the CLF model against the baseline measurement model to assess robustness.

The inclusion of the common latent factor resulted in a significant improvement in model fit (∆χ²(10) = 124.298, p < .001), suggesting that some common method variance is present in the data. However, as shown below, accounting for this variance did not compromise the structural integrity of the substantive factors.

**Fit Comparison between the Baseline and CLF Models**

| **Model** | **χ2 (df)** | **CFI** | **TLI** | **RMSEA** | **SRMR** |
| --- | --- | --- | --- | --- | --- |
| **Baseline CFA Model** | 175.939 (32) | .952 | .933 | .088 | .048 |
| **CLF Model** | 51.641 (22) | .990 | .980 | .048 | .028 |

Based on the squared standardized loadings from the CLF model, we partitioned the variance of the indicators into substantive variance (explained by the theoretical construct) and method variance (explained by the CLF). Average substantive variance is 57.3%, and average method variance: 28.6%.

While the method factor accounts for approximately 29% of the variance, this is within the range commonly observed in self-report behavioral research. Importantly, the substantive variance component (57.3%) remains the dominant driver of the model structure, being roughly double the magnitude of the method variance.

In addition, the critical test of common method bias is whether the theoretical factor loadings remain significant after controlling for method variance. As shown in the table below, while the standardized loadings were adjusted when partitioning variance, all substantive loadings remained statistically significant (p < .001) and substantively strong (ranging from .539 to .880). This confirms that the parcels represent their intended theoretical constructs beyond any shared method variance.

**Standardized Factor Loadings After Controlling for Common Method Variance**

| **Construct / Parcel** | **Substantive Loading (λ)** | **Method Loading** | **Significance (λ)** |
| --- | --- | --- | --- |
| **Digital Competency** |  |  |  |
| DC1 | .539 | .447 | p < .001 |
| DC2 | .841 | .585 | p < .001 |
| DC3 | .763 | .461 | p < .001 |
| **Digital Use** |  |  |  |
| DU1 | .665 | .550 | p < .001 |
| DU2 | .689 | .509 | p < .001 |
| DU3 | .880 | .556 | p < .001 |
| DU4 | .793 | .686 | p < .001 |
| **Creative Problem-Solving** |  |  |  |
| CPS1 | .799 | .496 | p < .001 |
| CPS2 | .798 | .521 | p < .001 |
| CPS3 | .751 | .497 | p < .001 |

Reference

Williams, L., Hartman, N., and Cavazotte, F. (2010). Method variance and marker variables: a review and comprehensive CFA marker technique. *Organ. Res. Methods* 13, 477-514. doi: 10.1177/109442811036603
